# Perceived Barriers to Gynecologic Care by Women Who Use Wheelchairs

**DOI:** 10.7759/cureus.15647

**Published:** 2021-06-14

**Authors:** Lauren Holt, Madeline H Carney, Lauren Duncanson, Christopher Hazen, Ambuj Kumar, Bri Anne McKeon, Laurie Woodard

**Affiliations:** 1 Family and Community Medicine, University of South Florida Morsani College of Medicine, Tampa, USA; 2 Family Medicine, University of South Florida Morsani College of Medicine, Tampa, USA; 3 School of Health Sciences, University of South Florida Morsani College of Medicine, Tampa, USA; 4 Biostatistics and Epidemiology, University of South Florida Morsani College of Medicine, Tampa, USA; 5 Obstetrics and Gynecology, University of South Florida Morsani College of Medicine, Tampa, USA

**Keywords:** women’s health, disabilities, female wheelchair users, gynecology, health care disparity

## Abstract

Objective

The aim of this study was to evaluate the current barriers associated with gynecologic care as perceived by women who use wheelchairs.

Methods

This qualitative study evaluated the barriers to gynecologic healthcare as described by female wheelchair users. We recruited English-speaking female participants aged 18 years and older who primarily used a wheelchair for mobility through flyer and email distribution. Interviews were conducted by three investigators using a semi-structured interview guide and recorded for transcription. Two investigators reviewed all transcriptions for accuracy which were then coded to identify emergent themes.

Results

The thematic saturation was achieved with 16 interviews. The most common barrier cited was transferring to the exam table (n=16). Women reported that their providers lacked knowledge and experience with women who use wheelchairs (n= 11).

Conclusion

There are many barriers to gynecologic care for women who use wheelchairs. Interventions are needed to improve accessibility to care for women who use wheelchairs.

## Introduction

There are over 53 million disabled individuals within the United States, with 13% identifying as having a mobility impairment [1]. Although the Americans with Disabilities Act (ADA) has been in place for over 30 years, individuals with disabilities still face significant barriers to healthcare and are at a higher risk of receiving inadequate care [2]. Women with a disability are more likely to be out of date with routine examinations including but not limited to breast cancer and cervical cancer screenings [2].

Many hypotheses exist as to why women with mobility impairments face barriers in achieving adequate healthcare. From the patient’s perspective, these barriers include inaccessible facilities, improper scheduling techniques, inadequate appointment length, lack of training for transferring the patient, and prior unpleasant healthcare experiences [3,4]. Fewer than a quarter of obstetrician-gynecologists (OB/GYNs) received any training on providing healthcare to women with disabilities, resulting in deficits in knowledge, skills, and attitudes in caring for this population [4]. Providers often assume women with disabilities are less sexually active, leading to improper counseling on reproductive healthcare [4]. Physicians who provide contraceptive counseling do so less often with patients with a physical disability than those with typical mobility [4].

Despite the disparate healthcare that this population receives, few studies exist that specifically address the barriers to care faced by female wheelchair users. While one such study interviewed women regarding their barriers to care, they also included barriers perceived by the providers [5]. The aim of this qualitative study was to identify and describe the current perceptions of female wheelchair users solely in order to more comprehensively characterize their experiences in obtaining gynecologic healthcare. We believe the findings from this study can be generalized to better serve women in wheelchairs seeking gynecologic care.

## Materials and methods

Participants

Participants were women who primarily use a wheelchair for mobility. These women were recruited via approved flyers distributed to known communities of wheelchair users as well as through referral by a family medicine physician (L.W.) and the ADA Coordinator at the University of South Florida. Inclusion criteria were as follows: (1) female; (2) primarily wheelchair users, either manual or electric wheelchair, (3) 18 years or older; (4) English speaking; and (5) able to provide informed consent. English speaking was used as an inclusion criterion as we were unable to provide translation services. No geographic constraints were used as an eligibility criterion in order to reach a wider and more diverse population of women. Participants were recruited between June 2019 and October 2019 via email and phone call. Each referring individual (L.W., ADA Coordinator) received permission to provide the investigators with the contact information of the participants. In addition, many participants referred other individuals from their communities to participate. Prospective participants were then contacted via telephone by one of three investigators (M.C., L.H., or L.D.) and informed about the study. An interview time, date, and location were then determined by the interviewer and participant. Informed written consent was obtained prior to the start of the interview. The first two interviews were conducted in person at private locations selected by the respective participants. In an attempt to eliminate the burden of transportation, and to combat any geographic constraints that prevented participation, subsequent interviews were conducted over the phone with both the participant and interviewer in secure and private locations. A total of 19 women were approached by our study staff, of whom three declined to participate because they did not want to disclose information regarding their gynecologic health. After interviewing 16 participants, it was determined that thematic saturation had occurred and therefore recruitment was ended.

Procedures

All procedures were performed in accordance with ethical standards of the Institute Research Committee. The study protocol was approved by the University of South Florida Institutional Review Board prior to study initiation. Three female, able-bodied interviewers conducted interviews after explaining the study, answering all questions, and obtaining written informed consent. At the time of the interviews, the interviewers (L.H., L.D., and M.C.) were medical students.

Interviews were conducted using a semi-structured interview guide based on the goals of the study with input from both an OB/GYN (B.M.) and family medicine physician (L.W.). The interview questions were designed to allow participants to share their experiences of receiving gynecologic healthcare. Interviews lasted approximately 45 minutes. No repeat interviews were carried out, and transcripts were not returned to the participants. No compensation was awarded to participants.

Qualitative analyses

Interviews were audio-recorded, transcribed verbatim by team members (L.H., L.D., and C.H.), and de-identified. Three of the authors (M.C., L.H., and L.D.) read all interviews and developed a codebook based on emergent themes. Using the preliminary codebook, two of the authors (either M.C. and L.H. or L.D.) then independently coded eight interviews at a time. Reviewers then met to discuss the coding of each interview and resolved coding discrepancies via consensus. All interviews were entered into ATLAS.ti. The number of times a code was used was counted and entered into a table.

## Results

A total of 16 women were interviewed in age ranging from 23 to 71 years, with an average age of 41 years and a median age of 39.5 years. Participants used a wheelchair for a wide variety of reasons including hereditary conditions such as Friedrich’s ataxia, traumatic spinal cord injuries, transverse myelitis, birth complications such as cerebral palsy, and progressive mobility impairments such as multiple sclerosis. The variety of conditions also provided a diverse sample as some women had been wheelchair users for their entire life, and others began using a wheelchair sometime later in their lives. All participants had previously seen a gynecologist or other healthcare provider for gynecologic health needs. The most common barrier cited was difficulty in transferring to the exam table (n=16). The second most common barrier was lack of provider knowledge about the participant’s specific disability (n=11). Other barriers to care cited by participants are outlined in Table 1. The findings were grouped by the most common themes: inaccessible medical equipment, difficulty in finding a provider, and provider and staff’s negative attitudes and lack of knowledge. Participants also described the components of a positive encounter with a physician providing gynecologic care.

**Table 1:** Barriers to Care Identified by Female Wheelchair Users OB/GYN, obstetrician-gynecologist

Number of participants who cited the barrier	Barrier to care
Accessibility	
16	Transferring to exam table
9	Difficulty in finding a provider
8	Participant feels they must inform provider ahead of time about their disability
7	Proper positioning on exam table
7	Participant has to travel far distances or go to many different providers to receive accessible care
7	Participant must bring an aid to appointment or is encouraged to bring an aid by staff/provider
5	Inaccessible exam equipment/office space other than table
5	Transport to appointment
4	Difficulty in positioning for mammogram
Provider knowledge/skills	
11	Provider lacks knowledge of patient's specific disability
9	Participant feels that provider lacks experience treating patients with disabilities
7	Participants feel it would be helpful to have a staff member close-by during exams to control spasms or help with balance
Attitudes	
10	Participant has to be the one to bring up sexuality/reproduction
9	Participant is discouraged and upset by her OB/GYN care
8	Participant feels they are not treated the same as able-bodied people
7	Provider assumes the participant is not sexually active
5	Participant stopped going for OB/GYN care due to bad experiences
When things go well	
14	Participant appreciates when the provider accommodates her needs (needs secondary to disability)
13	Participant appreciates when the provider is knowledgeable of her specific disability or has experience with patients with disabilities

 Inaccessible exam tables, rooms, and office space

The topic of transferring to the exam table for a pelvic exam was discussed at least once during every interview. Transferring to the exam table becomes problematic for these women given the considerable height difference between the exam table and the woman when she is seated in her wheelchair. Participant 2 commented that the exam table was “above her head.” She recalled another one of her experiences at her doctor’s office: “I would walk in and you would be looking at the nurse and they would be looking at you and you would be like ‘Ok, how are you going to get up there?’ and I don’t know how am I? [The tables] don’t elevate, they don’t go up and down. And a lot of the time the nurses don’t want to help because of liability reasons.”

Some women brought someone to their appointments who could assist them in transferring. Participant 6 commented, “And it was a good thing my mom was there, because she got me on the table. I couldn’t have gotten on the table without her.” However, not all patients have the ability to have a family member present. Participant 1 commented, “You can’t always bring a friend, there’s no ‘rent-a-friend' for the doctor’s office.” For some women, having to bring someone along represents a loss of independence. When discussing the topic of bringing an aid to the appointment participant 2 commented, “It makes you uncomfortable. So, for many years I did not go and did not want to go.”

In the absence of a table that lowers, many of the participants resorted to other uncomfortable solutions. Participant 7 described one of her experiences: “They didn’t have a table that lowered, so they had to lift me up, which I detest. It was kind of a critical thing and I needed to be seen so I had to be lifted because there was no other option.”

Seven women mentioned that even if they are able to transfer to the exam table, they have difficulty in positioning properly for the exam. This includes placing their feet into the foot rests and keeping them there during the exam. Six women mentioned feeling unsafe on the table. Participant 2 noted, “They always want you to scoot down and put your legs in the stirrups and I have always had a problem with that since my legs do not go 180.” Spasms can interfere with the exam and cause added discomfort. Women often noted that it would be helpful to have someone at their side to help maintain positioning which would diminish complications of spasms during the exam.

Five women reported that equipment other than the exam table was inaccessible. They noted that the exam rooms were too small to accommodate their wheelchair or the office did not have a wheelchair accessible scale. For some, even the front desk was too high for wheelchair users. Participant 12 said, “I’m usually shouting to the person about my personal insurance information instead of being able to actually be a little more private.”

This issue of proper positioning and transferring does not appear to be limited to the equipment required for a pelvic exam. Half of the participants were over the age of 40 years and eligible for screening mammography. Four of the eight women eligible noted difficulty in obtaining a screening mammogram. One woman recounted having to travel to three different imaging centers in one day attempting to get a mammogram.

For all participants, exam rooms, tables, and/or office equipment served as a barrier to obtaining proper care. Five of the participants reported that they stopped seeking gynecologic care altogether because of poor experiences related to such barriers.

Difficulty in finding a provider

All participants described difficulty in finding providers who had experience treating patients with disabilities. Some participants reported dedicating significant time into researching providers, as participant 12 told us, “…you basically have a list of the OB/GYN providers in your area, and you have to sit there and call and call and call and ask for this type of specific exam table.” Information regarding accessible facilities or experienced providers is not readily available. Participant 4 said, “[I was] referred to many, many different places before finally ending up with someone with spinal cord injury experience.”

Seven of the participants were able to find an experienced provider with accessible equipment but they had to travel significant distances in order to be seen in the clinic with accessible exam tables. Participant 2 reported her experience with this issue: “I am going to your location because it is close to my house and convenient but the table is above my head but the one [accessible] table that you do have is on the other side of town…So, I go now, sporadically, to the further away one, since you have to take off a whole day to get there and back basically.” In addition to traveling significant distances, five women reported transportation as a barrier to receiving care. Participant 8 described a negative experience she had with a provider and then added, “but I don’t know who else to go to,” when explaining why she continued care with that provider.

Provider/staff assumptions and negative bias

Participant 13 stated, “A lot of providers have the assumption that a lot of women with disabilities are not sexually active, so why even bother asking those questions? I’ve overheard nurses tell providers ‘Oh, by the way she is sexually active.’, and I don’t think they would do that to anyone else if they weren’t in a chair.”

Many women reported that their providers did not talk to them about their sex practices. During seven of the interviews, participants discussed feeling as if their provider assumed they were not sexually active. In addition, 10 of the 16 participants interviewed felt that they would need to be the one to bring the subject up to the doctor. Participant 8 said, “I know that my OBGYNs in the past just kind of assume that you are not sexually active, they don’t even broach the subject.” We asked her, “Do you think you were not asked about sexual activity because you use a wheelchair?” She replied, “Yes, I would assume that. Because I was asked that [about sexual history] before my injury.”

Sometimes these women found themselves being queried about their sexuality inappropriately. While participant 13 was sitting in a public setting for blood work to check for a sexually transmitted infection, she reported, “some of the medical assistants in the office weren’t used to a woman who used a wheelchair being in a relationship and having sexual relations, so they were asking me pretty general questions.” She expressed that she was happy to educate people, but was uncomfortable being asked so many personal questions publicly.

Although rare, some participants reported situations in which after they expressed their interest in sex and having children, they were met with hostile and inappropriate responses by medical professionals. After asking her doctor about getting pregnant, participant 2 was asked “Why would you want to have kids and bring them into this world when you already need help?” Two women identified a need for increased sex education for women with physical disabilities.

Lack of provider/staff experience and expertise

During the 16 interviews, nine participants stated that they felt that their provider lacked experience with patients with disabilities in general. Eleven women reported that the providers lacked adequate knowledge of their specific disability. When asked directly if she felt like their provider had adequate knowledge in working with her condition, participant 16 replied, “No. Nope. I don’t mean to be that blunt, but no. Most of them do not have any experience with any type of disability. Especially a physical one.”

Participant 12 stated that “I’ve never had a provider that has been knowledgeable about the fact that I have transverse myelitis, only the fact that I’m disabled. They have zero knowledge on spasms, or [that] I get bladder infections more frequently than other people because I do self-cath, and they don’t have knowledge on autonomic dysreflexia. So, I can’t use tampons because my body reacts to them. That’s a big deal…So, I’ve never had a good provider that has been knowledgeable about anything spinal cord related.”

Participants expressed that they did not expect every specialist to be an expert in their specific disabilities, but wanted them to do their due diligence and at a minimum read the chart before their visits. Participant 3 shared a story of having a procedure done by a physician covering for her usual gynecologist. The participant stated that after she was on the table, she felt that the provider “clamp down” and she flinched with pain. Her provider responded, “Oh wait, you can feel that? Wait, so you’re not paralyzed?” and “I said ‘no!’ so she saw the wheelchair and thought ‘paralyzed’.” She was frustrated that her provider did not take the time to learn about her past medical history and treat her appropriately. As a result of experiences like these, four women expressed that they felt as though they would never be able to find a provider to care for them.

When things go well

It is important to highlight that many of the women we interviewed had positive experiences to report. Fourteen of the women mentioned that they appreciate when their provider is accommodating to their needs. As participant 16 said in regard to finding a position for a pelvic exam, “We need to work together to see what different ways we could work this out.” One woman detailed at length how her provider does the pelvic exam slowly because she is familiar with how her spasms may interfere with the exam. She reported that this alleviates her anxiety and helps to make the appointment run smoother. Additionally, 13 women commented that they appreciate when their provider is knowledgeable regarding their specific disability or has experience working with women with disabilities. Participant 2 said that her provider researched her disability before they met for the first time. She commented, “She will sit down for more than the 5-10 minutes she is allotted and will go through things and I appreciate that.” While this study investigated the barriers to care, we were also able to highlight practices that are already being done well by gynecologic healthcare providers to ensure proper care for women who use wheelchairs.

## Discussion

Women with disabilities receive less preventative healthcare [3]. This study interviewed women who are wheelchair users for a variety of impairments and investigated barriers to gynecologic care from the patient’s point of view. While other groups have studied this issue, this is the first paper that provides solely patient testimony. By focusing on the patient’s perspective, the needs of this population of women are given the rightful and necessary platform for education and change. Additionally, our study uniquely quantifies the findings from most prevalent to least prevalent barriers, both physical and attitudinal, so that providers may enact change in ways that will benefit these patients the most.

From the moment a woman who uses a wheelchair tries to schedule an exam, she is faced with a system currently designed to accommodate those with typical mobility, which is not designed to accept her. Accessibility barriers begin with patients having to call multiple offices in order to locate one with an exam table that will accommodate her. Strategies for facilitating the transfer to the exam table such as exam tables or professional lift teams would alleviate the difficulty associated with the exam, as well as the need for women to bring an aid to appointments to assist with transferring. However, the table is simply a physical manifestation of the systemic prejudices that the physically disabled community faces in healthcare. From the patient’s perspective, an inaccessible table represents that the healthcare community marginalizes and dismisses this population’s gynecologic healthcare needs. Interestingly, there is sufficient literature to suggest that the healthcare system is working to understand how to overcome this community’s barriers to obtaining healthcare. This disparity between patient’s perceptions and the healthcare community’s efforts may represent the lack of communication between the two groups. Access to care, both gynecologic and otherwise, may be improved by more open communication between provider and patient.

The gynecologic healthcare needs of women with disabilities are not well established, as the American College of Obstetrics and Gynecology (ACOG) has not offered broad practice guidelines on caring for this population and only for adolescents with physical disabilities [5]. The paucity of recommendations for caring for this population may be reflected in how many women feel their providers lack knowledge of their specific needs, as reflected in this study. Additionally, inaccurate perceptions of people with disabilities by physicians, reflecting biases of the general public, may be then augmented by the lack of formal training in caring for women with disabilities. One study showed that out of 500 OB/GYNs, only 17.2% had received any information or training on providing healthcare to women with disabilities [4]. Participants also feel marginalized by other office and medical staff, who likely experience the same educational gap as physicians. Increasing training and exposure to those with different disabilities during medical school, residency, and other medical training programs will not only help improve the lack of experience but also help challenge the implicit and explicit biases that currently exist. Many training resources are already available, and it is up to the medical community to ensure they are being utilized.

Women who use wheelchairs continue to be perceived as asexual despite the fact that many women with disabilities engage in consensual and fulfilling sexual activity. Gynecologists are an important resource for sexuality education [6]. Physicians providing gynecologic care need to be equipped to provide such education for all women, including those with disabilities. Providers and staff must be knowledgeable of the fact that all humans are sexual beings, regardless of mobility status. OB/GYNs are less likely to initiate contraceptive counseling for patients with disabilities despite that this population has similar needs for contraception, as reflected in our study [6]. This further emphasizes the implicit biases held by medical providers that women with physical disabilities are asexual. It is imperative to avoid assumptions regarding the sexuality of women who use wheelchairs.

Barriers to healthcare for persons with physical disabilities is not limited to the gynecologic setting. On review of the literature on care for this population in the primary care setting, many of the same barriers have been reported. However, solutions to these barriers are not acceptable when applied to gynecologic care. One study showed that 76.2% of primary care providers performed the physical exam with the patient remaining in their wheelchair [7]. While some aspects of the physical exam may be adequately performed in a seated position (i.e., ear, nose, and throat exam), a complete pelvic exam necessary for women’s health visits is simply not feasible. The study also indicated that 52.4% of providers asked the patient to bring someone with them to the appointment for assistance, which we heard as a common practice from many of our participants as well. Unfortunately, 44.4% of the providers in the study chose to skip portions of the exam due to physical barriers [7].

Despite the novelty of the information provided, this study has limitations. First, the study population was comprised of volunteers interested in being interviewed and not a true random sample. This may have introduced reporting bias whereby patients who were unhappy or had negative experiences were more likely to participate. Additionally, many of the study participants were referred by physicians, and thus it was known that they were receiving some form of care. Research has shown that women with physical disabilities are less likely to receive routine cancer screenings. However, most of the participants in this study were up to date on these screenings. For these reasons, this study population may not accurately reflect the level of care received among all female wheelchair users. Lack of funding for the study was a major determinant of sampling protocol and prevented the investigators from organizing a larger and more in-depth study. However, the group of women interviewed clearly reflects at least a significant experience of women who have mobility impairments and are primarily wheelchair users.

Based on the barriers elicited in this study, we have developed recommendations for the medical community in providing robust care to women who use wheelchairs. Every office should have at least one chair that can easily change position for patients who use wheelchairs. Patients who require use of this chair should be scheduled to ensure that the room with the chair will be available at the time of her appointment. Providers should also communicate openly with their patients so that they understand the specific needs of each woman. For example, a provider should be aware of the patient’s underlying condition that requires her to use a wheelchair. We recommend implementing a more standardized curriculum in training OB/GYN residents in regard to caring for women with disabilities. Lastly, there should be a baseline understanding by all gynecologic care providers that women who use wheelchairs may have unique needs when it comes to healthcare but are human and deserve to be treated as such.

## Conclusions

There is a paucity of information describing how to care for women who use wheelchairs and why they continually receive disparate healthcare. Of the data available, the perception of the patients themselves is obscured by those of the medical providers. It is imperative to receive input from the population served. As widely said in the disability community: “Nothing about us without us." By highlighting the most common barriers to proper care, as reported by the women themselves, we can begin to eliminate the glaring inequalities that persist for this population.

## Appendices

Appendix 1: Semi-Structured Interview Guide

Thank you again for your willingness to participate in this interview. I want to remind you that your participation is completely optional, and if at any point you no longer wish to participate, the interview will be terminated immediately without penalty. As a reminder, the goal of this project is to investigate barriers that exist between female wheelchair users and gynecologic healthcare. During this interview, you will be asked to talk about your knowledge, experience, and attitudes surrounding your gynecologic health and any experiences you have had regarding this. The goal of this project to address the concerns and improve the relationship and overall care of women who use wheelchairs and providers of gynecologic healthcare.

1.  For how long have you been using a wheelchair?

2.  Do you see a provider for gynecologic care/“women’s health”? (i.e., paps, mammograms, sexual performance, counseling, abortion, fertility, family planning?)

a.  If yes, what provider do you most often visit for this care? A gynecologist, your primary care doctor, an ARNP (advanced registered nurse practitioner), etc.?

3.  When was the last time you received gynecologic care? How often do you go?

4.  Do you have insurance?

5.  Tell me about an experience you have had with your OB/GYN or gynecologic healthcare provider (positive or negative)

a.  Is there something that happened that you liked or didn’t like?

6.  What are your reproductive goals?

a.  Are you sexually active?

b.  Do you have any children/do you intend to have children?

c.  Has your OB/GYN (or other physician that they have previously specified) supported your goals?

d.  If not, how do you feel your provider could best support you?

7.  Does your OB/GYN or other gynecologic healthcare provider talk to you about safe sexual activity and family planning/birth control when you visit them?

a.  Do you bring it up to them? Or do they bring it up to you?

b.  How does this conversation usually go?

8.  Does your provider talk to you about menstruation?

9.  Have you had a mammogram?

a.  Were there any additional accommodations?

10.  Have you had a pap smear?

a.  Were there any additional accommodations?

11.  Are there things you wish you could talk to your OB/GYN or gynecologic healthcare provider about but either do not feel comfortable or feel that you are unable to ask?

12.  Logistics of the visit

a.  What, if any, issues did you encounter?

b.  Did you experience any difficulties with the set-up of the exam room?

i.  If clarification needed: For example, maneuvering around the room, getting onto the exam table, etc.

c.  Did you receive assistance with transferring? (if applicable)

d.  Did you or your provider’s office address ahead of the visit that you use a wheelchair?

i.  Were any accommodations made?

13.  Did you feel that your doctor was accommodating and understanding of your mobility?

a.  How so?

14.  What do you feel could make visiting your gynecology provider easier?

15.  Do you know how often it is recommended that women receive gynecologic care?

If they DO NOT receive gynecologic care

1.  Where do you get information regarding your gynecologic and reproductive health?

2.  What is preventing you from going to a provider for your gynecologic healthcare?

a.  What would need to change to get you to go?

3.  When you do visit your doctor (of any specialty) are there things you particularly like that they do to accommodate your needs? Anything you dislike?

Is there anything else surrounding these topics that we have not discussed that you would like to share?
